# An analysis of the psychometric properties of the writing-specific cognitive strategies questionnaire for undergraduate students

**DOI:** 10.3389/fpsyg.2023.1274478

**Published:** 2023-11-16

**Authors:** Olga Arias-Gundín, Patricia Robledo

**Affiliations:** Department of Psychology, Sociology and Philosophy, University of León, León, Spain

**Keywords:** writing strategies, questionnaire, undergraduate students, psychometrics, validity

## Abstract

Writing strategies are needed to manage the complexity of writing tasks, especially at university, where writing tasks are for learning, professional, or scientific purposes and are highly demanding. The literature shows that many undergraduate students have defined, stable, writing strategies, although some lack proper strategic development and require explicit instruction in this regard. In both cases, adapting writing tasks to undergraduate students’ preferences and instructing them effectively requires understanding their writing strategies, which will encourage optimal learning and writing proficiency. This is why valid, reliable, writing strategy assessment tools are essential. The present study focused on the validation of the Spanish Writing Strategies Questionnaire-Undergraduate Students (WSQ-SU), aimed at measuring undergraduate students’ preferences for using different writing strategies. The sample comprised 978 Spanish undergraduates doing degrees in Infant, Primary or Social Education, Pedagogy and Psychology. The data from the questionnaire was explored by means of exploratory and confirmatory analysis, test–retest reliability to analyse temporal stability and convergent validity. Two factors, planning and revising, were identified through exploratory and confirmatory factor analysis, representing different writing strategies and supporting the original model. The results indicated adequate test–retest reliability and temporal stability. The results also showed the questionnaire’s convergent validity; a direct, linear correlation between two factors and off-line planning and revising variables. Based on the results, the WSQ for undergraduate students-Spanish version has been shown to be a reliable and valid, scale that can be easily applied in the university context to explore undergraduate students’ writing strategies.

## 1 Introduction

Mastery of written composition is key to people’s academic, professional and social success, and during education, writing is a basic tool both for learning other subjects and for demonstrating what has been learned (Graham and Hebert, 2011; Graham et al., 2015). Similarly, in today’s information and knowledge society, how people write has an impact on their employment possibilities and advancement, and is essential in qualified professions that require written text (National Commission on Writing, 2005, 2006). In addition, at a social level, people’s active participation in the information and communication technology (ICT) society increasingly demands the use of writing as a tool for communication and socialisation (National Commission on Writing, 2005; Olson and Oatley, 2014). In other words, writing competence is a tool for learning, professional development and socio-personal communication, and is essential for personal development and fulfilment, active citizenship, social inclusion, and employment (National Commission on Writing, 2006; Graham, 2018). Consequently, one of the main purposes of the education systems in the international context, including Spain, is to promote students’ written communicative competence, from initial educational stages (Spanish Education Law, LOMLOE, 2020) to university (Castelló and Castell, 2022).

Writing is a problem-solving task that places a lot of cognitive demands on the writer (Hayes, 1996), in other words it is a complex activity that takes effort. Mastering writing, in addition to automating transcription skills, requires self-regulation of high-level cognitive processes (Graham and Harris, 2000). According to the different theoretical writing models, these high-level cognitive processes refer to planning, which involves generating ideas and organising them in a textual structure following an established plan; translating, which involves transforming these ideas into a written text, including transcription skills; and the revision process, which involves reading the text and evaluating it according to the established plan in order to identify errors and edit them through any necessary corrections (Hayes and Flower, 1980; Hayes, 1996, 2011, 2012; Kellogg, 1996; Berninger, 2000; Berninger and Winn, 2006). Self-regulation of high-level cognitive processes is a critical aspect of writing and it is represented by the use of writing strategies (Zeidner et al., 2000; Santangelo et al., 2016; Puranik et al., 2019), since proper activation of planning and textual revision processes contribute to achieving higher quality written texts (Beauvais et al., 2011; Limpo et al., 2014; Limpo and Alves, 2018).

Managing the complexity of a writing task makes a writing strategy necessary (Torrance and Galbraith, 2006). Writing strategies allow writers to regulate the attention they pay to the writing processes and contribute to reducing cognitive overload (Kieft et al., 2006; Beauvais et al., 2011). Empirical research has shown that writers’ strategic behaviour during composition strongly predicts the quality of “novices’” and “experts’” texts (Beauvais et al., 2011; Graham et al., 2017, 2019; Wijekumar et al., 2019). Accordingly, the use of writing strategies has generally been considered a critical individual writing-related variable (Kieft et al., 2008) and is a major focus of research in writing instruction (Harris et al., 2010; Graham and Harris, 2018) from the earliest stages of education (Arrimada et al., 2019) to university (MacArthur et al., 2015; Mateos et al., 2018; Granado et al., 2019; Lammers et al., 2019; MacArthur and Philippakos, 2022).

Writing strategies are understood as the way people tend to organise cognitive processes such as planning or revising (Kieft et al., 2006) or the sequence in which a writer plans, composes, revises and does other writing related activities (Torrance et al., 1999). Two dimensions are usually used to describe the differences between writing strategies. The first is related to how much writers tend to plan before writing, the second is about how much writers tend to rewrite and revise their texts. Students who follow a planning strategy prefer to have clarified their ideas before starting to write, so that they first think about and decide on content and organisation before writing, making a few drafts. Writers who prefer the revising strategy tend to use revision to develop the content of the text. Students with this profile first write a rough draft and then revise it; writing helps them to clarify their ideas and better understand their own arguments. They are students who think while they write, so they tend to do multiple drafts (Galbraith and Torrance, 2004). There are also writers with a mixed profile, some with similar scores in both types of strategy who plan the content before producing the text, but who change it in subsequent revisions (Torrance et al., 1994). Others make little use of either strategy, producing poor quality texts with little development (Torrance et al., 1999).

University students do a lot of writing. Undergraduates have to write for academic, scientific and professional purposes (Castelló and Castell, 2022). Their writing tasks usually consist of analysing different sources of information on a subject and preparing a new written document from those sources, comparing, transforming and integrating ideas in a connected, organised way. Such synthesis tasks are hybrid activities that require writers to select information, connect it, and organise it within a new textual structure to produce new, original written discourse (Spivey and King, 1989; Spivey, 1997; Perin, 2013). Synthesis is a complex writing task requiring the mediation of planning, monitoring and reviewing strategies throughout the whole process (Flower and Hayes, 1980; Castells et al., 2023; Valenzuela and Castillo, 2023). Consequently, the writing tasks required at university are complex, with high demands on students’ processing and cognitive activity. As noted above, to manage the many constraints, writers need to organise the cognitive activities involved in writing and appropriately activate writing strategies. Several studies have attempted to explore how undergraduate students vary in their use of different writing strategies. Those studies have identified differences in the use of writing strategies among undergraduate students and have also confirmed that many undergraduates’ strategic writing profiles are relatively well-defined and demonstrate some stability over time and in relation to the different writing tasks (Levy and Ransdell, 1996; Biggs et al., 1999; Torrance et al., 1999, 2000; Robledo et al., 2018). From an applied or pedagogical perspective, it is essential to know what writing strategies undergraduate students use, so that writing tasks can be tailored to their abilities, and thus really contribute to learning. Determining students’ writing strategy preferences can be an intermediate step on the way to identifying their (categorical) writing profiles and this is important as offering students writing tasks that match their writing profile may have a positive impact on their domain learning and may help reduce the cognitive load of writing, because planning or revising writing strategies allow the content of the text to be planned free from the demands of constructing well-formed, coherent texts (Torrance and Galbraith, 2006). Apparently, the closer the demands of the written composition task to students’ writing strategies, the better their performance (Kieft et al., 2008). There is a clear pedagogic benefit to developing an understanding of undergraduates’ writing strategies. However, it is important to bear in mind that a proportion of high school students do not have fully developed, persistent writing strategies when they enter university (Kieft et al., 2006). This complicates their ability to successfully cope with the writing tasks required at this educational stage, and in consequence, many undergraduates have difficulties with writing. This may have an impact on their academic achievement, professional career, and overall participation in society (Boscolo et al., 2007; Mateos and Solé, 2009; Cumming et al., 2016; Konstantinidou et al., 2023). These students require specific, explicit instruction in written composition to help them acquire—or refine and adapt their own—strategies in order to effectively deal with academic, scientific, and professional writing tasks (MacArthur et al., 2015; Wischgoll, 2017; Graham and Harris, 2018; MacArthur and Philippakos, 2022).

All of this underscores the need to determine what writing strategies undergraduates use and what strategies they lack, especially bearing in mind that they are no longer novice writers, having accumulated considerable experience of writing through schoolwork and exams, but have typically yet to develop expertise to match that of successful professionals (Torrance et al., 2000). Doing that requires valid, reliable assessment tools. Previous studies in this field used data collected through scales or questionnaires, which may have led to biases due to self-reported estimates of writing strategies (Fidalgo and García, 2009). However, researchers using self-reporting showed that it was possible to detect individual differences between writers with self-reporting writing questionnaires (Torrance et al., 1994, 1999, 2000; Galbraith, 1996, 1999; Lavelle et al., 2002). In addition, similar results have been found by comparing questionnaire data with data obtained through online or retrospective measurements, where writers describe their writing activities during the writing process (Torrance et al., 1999). This confirms the functionality of the questionnaires for assessing students’ writing strategies. In addition, questionnaires it is a feasible alternative for exploring writing strategies which would allow researchers to collect data from a representative sample size. However, the writing strategy questionnaires found in the literature review have limitations. Some of them assess general cognitive, metacognitive and/or motivational strategies applied to the field of writing, but not specific writing strategies linked directly to the core cognitive processes of writing, such as planning and revision (Lavelle et al., 2002; Raoofi et al., 2017). Other questionnaires have been designed and validated to evaluate writing strategies when writing in a second language, a task that demands the activation of some different cognitive processes from those required by writing in the mother tongue (Petrić and Czárl, 2003). In some cases, the scales used have not been subjected to empirical validation processes, or at least these data are not reported in the papers (Torrance et al., 1994, 1999, 2000). Furthermore, previous studies using the questionnaire that the present study assesses—examining two writing specific strategies: *planning* and *revising*—only looked at primary and secondary education (Kieft et al., 2006, 2008; Arias-Gundín et al., 2021). This means that the questionnaire has not been validated in a Spanish-speaking university sample. Furthermore, the data from those studies produced differing results about the factorial structure of the scale, meaning it was not possible to confirm it clearly. This underscores the importance of examining the validity and psychometric properties of writing strategies questionnaires. The benefits of exploring these aspects of questionnaires about writing-specific strategies include the possibility of capturing students strategy preferences non-intrusively, exploring some aspects that remain unclear about undergraduate students’ writing abilities (i.e., strategy stability), which will later allow consistent writer profiles to be established based on the core cognitive processes of writing (planning and revision) and the possibility of comparing undergraduate student outcomes according to their writing strategy preference in instructional studies as one key individual feature of adult writers.

Therefore, the main goal of this study is to analyse the validity and the factor structure of a Spanish writing-specific strategies questionnaire for Undergraduate Students (WSQ-SU) (Kieft et al., 2006, 2008), analysing the fit of the proposed factorial model based on the traditional two-factor model: Planning and Revision (Kieft et al., 2006, 2008). The second goal of the study is to analyse the temporal stability of the questionnaire, analysing test–retest reliability. The third goal of the study is to analyse the questionnaire’s convergent validity by examining the correlation between the questionnaire’s writing strategy factors and off-line planning and revising measures in a synthesis task. The research question that this study aims to answer is: Does the WSQ-SU questionnaire have stable psychometric properties and a factorial structure that fit the classic cognitive models of writing, allowing valid, reliable identification of planning and revising in Spanish-speaking undergraduate students?

## 2 Materials and methods

### 2.1 Participants

The study sample consisted of 978 Spanish undergraduate students from two different Spanish Universities, obtained through convenience sampling. Most of the sample (71.38%) were women (*n* = 696) and 28.62% were men (*n* = 286). They were studying various degrees: Infant Education (*n* = 477), Primary Education (*n* = 295), Social Education (*n* = 138), Pedagogy (*n* = 32) and Psychology (*n* = 36). This sample allowed us to address the different study aims in three phases. Table 1 shows the distribution of the sample by gender, degree, and study phase.

**Table 1:** Distribution sample of study 1 by course year, gender, and degree.

	First phase		Second phase		Third phase	
	Male	Female	Male	Female	Male	Female
Infant education	58	174	69	176		
Primary education	79	137			16	63
Social education	11	34	36	57		
Pedagogy					7	25
Psychology					6	30
Total grade	493		238		147	

In the initial study phase—to analyze the factor validity of the questionnaire—the full sample was used, meaning that 978 undergraduate students were asked to complete the questionnaire during a 15-min period at the beginning of class. One year later, in the second phase of the study, a subsample of 94 Infant Education students (84 women and 10 men) were asked to complete the same questionnaire again. This allowed us to analyze stability of the questionnaire over time. Finally, in the last phase of the study—to analyze the questionnaire’s convergent validity—103 university students (73.79% women and 26.21% men) were asked to complete the WSQ-SU and write a synthesis text from two source texts in a 90-min session. The majority (70.80%) were studying for a degree in Primary Education, and 29.29% were studying for a degree in Infant Education.

### 2.2 Procedure

The method used to translate the questionnaire was translation and back-translation of the original questionnaire by native speakers (Hambleton et al., 2005).

Data was collected with the consent of the teachers in each subject. All students participated voluntarily after providing verbal informed consent. They completed the WSQ-SU in the classroom and personal identifying data was not recorded. One of the study researchers was present during the application of the questionnaire to answer any questions the students may have had. The three phases of the study were conducted following the World Medical Association Code of Ethics (Declaration of Helsinki) (Williams, 2008).

In phase 1, all of the students in the sample were asked to complete the questionnaire during a 15-min period at the beginning of class. In phase 2, a subsample of the initial sample was asked to complete the same questionnaire again 1 year later. Finally, in phase 3 another subsample of the initial sample was asked to complete the WSQ-SU and write a synthesis text from two source texts in a 90-min session.

### 2.3 Measures

The Writing Strategies Questionnaire (WSQ-SU, see Supplementary material) is a scale with 26 items (Kieft et al., 2008). The original version examined two writing strategies with high-school students: *planning* (11 items) and *revising* (15 items). Students rate their agreement with each item on a five-point scale from 1 (*completely disagree*) to 5 (*completely agree*).

In order to assess off-line planning and revising, students were asked to write a synthesis text. They were given a piece of paper for their rough draft, another for their final text, and two source texts with scientific information related to the following topics: “ICT in university education” and “students’ free time.” *Taking notes* was measured using a scale ranging from 0 (there are no notes) to 2 (well-developed notes, whether the notes add new information, transform the ideas of the source texts, synthesise extensive information in keywords…). *Idea generation* analyses how students generate ideas using a scale from 0 (there is no draft) to 3 (the ideas appear in a list without any order and apparently unconnected). *Effective revision* is the number of effective changes made by the student from the draft to the final text. Each variable was scored by two independent raters who had been trained. The reliability of the measure was acceptable (taking notes 0.96; idea generation 0.93; effective revision 0.88).

### 2.4 Data analysis

In each study phase, kurtosis and skewness were assessed, with indices within ±1 indicating normal distribution (Valdés et al., 2019). Data analysis was performed using SPSS and AMOS version 24 software.

#### 2.4.1 Phase 1

Factor validity was determined by exploratory factor analysis (EFA), which supported the original structure of the instrument in the Spanish version. Maximum likelihood estimation methods were used and the input for each analysis was the item covariance matrix.

#### 2.4.2 Phase 2

A test–retest study was conducted to analyse the temporal stability of the questionnaire. The correlations between the mean scores from the two evaluation timepoints were assessed using Pearson’s correlation. In addition, a confirmatory analysis of the model was performed at test–retest time points. The model’s goodness-of-fit was evaluated using absolute indices—Chi-squared (*χ*^2^) with its degrees of freedom (*df*) and the Root Mean Square Error of Approximation (RMSEA)—and relative indices—the Comparative Fit Index (CFI) and Incremental Fit Index (IFI). The following rules were used to evaluate the model’s goodness-of-fit: the ratio of chi-squared to degrees of freedom (*χ*^2^/*df*) is lower than 5; CFI and IFI values above 0.90 are acceptable, and values below 0.08 for RMSEA are indicative of an acceptable fit (Collier, 2020).

#### 2.4.3 Phase 3

Convergent validity was explored with Pearson’s correlations between the questionnaire’s writing strategies factors and the off-line planning and revising measures in the synthesis task.

## 3 Results

Results are structured according to the stages of the research process. The findings from phase 1 (Exploratory and Confirmatory Factor Analysis), phase 2 (Temporal Stability), and phase 3 (Convergent Validity) are described below.

### 3.1 Study phase 1: exploratory factor analyses

As Table 2 indicates, all items demonstrated values within the range of normal distribution. The results from the KMO test, 0.76, and Bartlett’s test of sphericity, *χ*^2^ (66) = 2030.75, *p* < 0.000, support the suitability of the data for use in exploratory factor analysis (EFA).

**Table 2:** Writing strategies questionnaire item analysis.

Item	*M*	*SD*	Min.	Max.	Sk	K	α_F_	α_Q_	^*^Factor 1 planning	^*^Factor 2 revising
1. When I write a text, I spend a lot of time on thinking how to approach it.	3.41	0.85	1	5	0.01	−0.21	0.70	0.66	0.56	
2. I always use an outline before I start to write.	2.44	1.21	1	5	0.47	−0.58	0.66	0.66	0.69	
3. Before writing a text, I jot down some notes on a separate piece of paper. Later, I elaborate on these notes.	3.33	1.11	1	5	−0.25	−0.72	0.63	0.64	0.75	
4. Before I start to write a text, I prefer to write down some thoughts on a separate piece of paper to discover what I think about the topic.	3.00	1.22	1	5	−0.50	−0.97	0.65	0.65	0.70	
^R^5. Planning a text is not useful for me.	3.98	1.07	1	5	−0.79	−0.20	0.70	0.67	0.59	
11. I have to have my thoughts clear before I’m able to start writing.	3.96	0.86	2	5	−0.46	−0.49	0.71	0.68	0.50	
18. When I reread and rewrite my text, the structure of my text changes a lot.	2.79	0.91	1	5	0.41	−0.08	0.58	0.66		0.74
21. When I rewrite my texts, the content often changes a lot.	2.55	0.84	1	5	0.54	0.30	0.58	0.67		0.77
22. When I reread my texts, sometimes they are very chaotic.	2.47	0.95	1	5	0.41	−0.29	0.60	0.68		0.65
23. I have to reread the texts I wrote, to prevent redundancies.	3.67	1.02	1	5	−0.52	−0.26	0.68	0.68		0.34
26. When I am finished writing, I reread and improve a lot: I might change a lot in my text.	3.18	0.94	1	5	0.02	−0.38	0.59	0.65		0.67

Sk, skewness; K, kurtosis. ^R^Items recoded in the analyses. α_F_, change in Cronbach’s alpha of the factor the item belongs to if the item is removed; α_Q_, change in Cronbach’s alpha of the questionnaire if the item is removed. ^*^Factor loadings in EFA.

We retained the same two factors as in the original scale. The first factor, *planning strategy* has six items, reliability via Cronbach’s alpha of 0.72, and explains 23.42% of the variance. The second factor, *revising strategy* has five items, a Cronbach’s alpha of 0.70, and explains 17.84% of the variance. Each item only loads on a single factor, no cross-loadings were kept. Fifteen items were excluded because they did not fit the different factors (items 6, 7, 8, 9, 10, 12, 13, 14, 15, 16, 17, 19, 20, 24, 25). The resulting 11-item scale had a Cronbach’s alpha of 0.71 and explained 41.26% of the variance. Table 2 shows the factor loading and change in Cronbach’s alpha of the factor and the questionnaire if the item is removed, for each questionnaire item.

### 3.2 Study phase 2: temporal stability

This study used a subsample of Infant Education undergraduates one year after study 1. It produced adequate test–retest reliability indices (see Table 3): planning factor *r* = 0.50 (*p* < 0.001) and revising factor *r* = 0.46 (*p* < 0.001).

**Table 3:** Writing strategies questionnaire Item analysis at test–retest time-points.

	Test					Retest				
Item	*M*	*SD*	Sk	K	α_F_	*M*	*SD*	Sk	K	α_F_
Planning factor					0.71					0.76
1.	3.14	0.89	0.10	−0.15	0.70	3.63	0.72	0.34	−0.50	0.74
2.	2.64	1.25	0.32	−0.95	0.67	2.55	1.14	0.33	−0.67	0.73
3.	3.38	1.22	−0.23	−0.97	0.61	3.43	1.08	−0.50	−0.23	0.68
4.	3.00	1.23	−0.11	−0.99	0.67	3.24	1.24	−0.22	−1.10	0.70
^R^5.	3.91	1.05	−0.39	−1.19	0.70	4.05	1.08	−0.68	−0.75	0.73
11.	3.89	1.02	−0.70	−0.04	0.68	3.99	0.83	−0.43	−0.47	0.76
Revising factor					0.78					0.76
18.	2.83	1.00	0.22	−0.39	0.71	3.07	0.92	0.36	−0.44	0.68
21.	2.60	0.93	0.41	0.06	0.71	2.69	1.05	0.36	−0.21	0.68
22.	2.64	1.08	0.29	−0.70	0.74	2.54	1.05	0.45	−0.38	0.79
23.	3.69	0.93	−0.50	−0.16	0.82	3.83	1.01	−0.67	−0.05	0.70
26.	3.15	1.03	0.30	−0.40	0.73	3.38	1.03	−0.10	−0.72	0.69

Sk, skewness; K, kurtosis. ^R^Items recoded in the analyses. α_F_, change in Cronbach’s alpha of the factor the item belongs to if the item is removed.

In addition, to check that the model’s effectiveness was not significantly affected by time, it was subjected to CFA with the sample from the second time point. As Table 4 shows, the model, in the second phase of study, had an acceptable fit to the data according to indexes evaluated, as in the previous phase of study.

**Table 4:** Goodness of fit indexes for model proposed at test–retest time-points.

Model (*n*)	*χ*^2^/*df*	CFI	IFI	RMSEA (IC 90%)
Phase 1 (978)	4.68	0.90	0.90	0.06
Phase 2 (94)	2.12	0.90	0.89	0.08

### 3.3 Study phase 3: convergent validity

We calculated Pearson’s correlations between the questionnaire’s planning and revising factors and off-line measures: notes and idea generation (with planning) and effective review (with revising). The results show a direct, linear correlation between the factors and off-line variables. The planning factor demonstrated moderate correlation with both notes (*r* = 0.32, *p* < 0.05) and idea generation (*r* = 0.40, *p* < 0.01). There was a similar level of correlation between the revising factor and effective revision (*r* = 0.34, *p* < 0.05).

## 4 Discussion and conclusions

The goal of the present study was to analyse the factor structure and validity of the Spanish Writing Strategies Questionnaire for Undergraduate Students. Two additional goals were to analyse the temporal stability of the questionnaire and convergent validity.

In terms of factor structure, the first goal of our study, the results were in line with the previous study using the original version of the questionnaire that identified two factors: planning and revising (Kieft et al., 2006, 2008). This bifactorial structure for the WSQ-SU was identified in the initial exploratory analysis with a large sample of undergraduate students and was confirmed by one additional confirmatory factorial analysis—with a different sample, a year after the first study. Therefore, we can conclude that the WSQ-SU presents a clear factorial structure that allows identification of undergraduate students’ use of planning strategies or text revision. The factorial model proposed and validated in this study conforms to the classic theoretical assumptions of writing, which recognise the planning and text revision processes as two key core processes for learning and mastering writing (Hayes and Flower, 1980; Hayes, 1996, 2011, 2012; Kellogg, 1996; Berninger, 2000; Berninger and Winn, 2006), the management of which demands self-regulated implementation of writing strategies (Graham and Harris, 2000; Zeidner et al., 2000; Santangelo et al., 2016; Puranik et al., 2019). In addition, the proposed model’s bifactorial structure is consistent with previous studies in the field of writing strategies that have identified two common strategic profiles in writers, one of a planning nature and the other of a revising type (Torrance et al., 1994, 1999; Galbraith and Torrance, 2004; Kieft et al., 2006, 2008). However, our study indicates that the WSQ-SU—with 26 items in its original form—is more robust if some items that do not clearly saturate any of the factors are eliminated. The model proposed in our study offers a final scale with 11 items and presents adequate reliability. This is in line with a previous study using the questionnaire in its original English version confirming its efficacy and functionality with fewer (10) items (Kieft et al., 2006). However, a detailed analysis of both studies would be needed in the future, analyzing the content of the items that do not saturate on any factor to see if they are the same in both linguistic contexts. Furthermore, it would be advisable to restate the theorem that supports the proposal of a bi-factor scale that assesses planning and revising. In this case, it is important to bear in mind the fact that the planning and revising processes are recursive and can occur before or during translation, so they can be split into multiple subprocesses: online and advance planning, post-translation and online revision (Berninger and Swanson, 1994). These subprocesses seem to develop at different rates, so that as writers develop, they are more able to activate the most complex subprocesses (advanced planning and post-translation) (Berninger et al., 1992; Berninger and Swanson, 1994; Berninger et al., 1996). The factorial structure of the questionnaire that was proposed in the theoretical model that supports the present study could be complemented with some other factor that addresses the specific subprocesses within planning and revising, as has been demonstrated in studies with primary students in which the questionnaire exhibits a four-factor structure (Arias-Gundín et al., 2021).

The results related to the second study objective, assessing the temporal stability of the questionnaire, indicate adequate test–retest reliability indices for the planning and revising factors. This confirms that the questionnaire allows valid, reliable and stable identification of undergraduate students’ use of planning and revising strategies. This means that there is now a tool for evaluating appropriate writing strategies in the Spanish-speaking context, complementing other versions of the questionnaire that have been validated with students in primary education (Arias-Gundín et al., 2021).

Finally, the results in relation to the third study objective allow us to conclude that the questionnaire presents adequate convergent validity. There was a correlation between the planning and revising indices offered by the questionnaire and the specific planning and revising measures obtained off-line from the analysis of the activation of these processes by students while performing specific writing tasks (in this case, synthesis tasks). These results are in line with results from previous studies demonstrating the potential of questionnaires to, on the one hand, effectively measure the writing strategies used by students—offering data similar to that offered by other types of off-line and on-line assessment tools (Torrance et al., 1999, 2000)—and on the other hand, detect individual differences between writers with self-reporting writing questionnaires (Torrance et al., 1994, 1999, 2000; Galbraith, 1996, 1999; Lavelle et al., 2002).

Development of reliable measurement tools in all scientific fields has a critical prior step: validation (Muñiz and Fonseca-Pedrero, 2019). The major contribution of this study is the validation of the Spanish Writing Strategies Questionnaire for Undergraduate Students, indicating that it is a suitable tool for reliably and easily assessing undergraduate students’ writing strategies. Validation of the Spanish Writing Strategies Questionnaire for Undergraduate Students is a first step toward reliable analysis of undergraduate students’ use of writing strategies in the context of university writing tasks. This will continue with the analysis of aspects that have not yet been examined such as the interaction between undergraduates’ use of strategies and other key writing-related variables in dealing with writing tasks in the university context, and the moderating effect of writing strategies on synthesis task strategic instruction in university classrooms. In addition, because undergraduate writing behaviour can be adaptive and because undergraduates can vary their strategies or develop progressively more sophisticated strategic profiles depending on demands of tasks or contexts (Torrance et al., 2000), it would also be interesting to analyse how sensitive the WSQ-SU is in identifying possible variations in undergraduates’ writing-strategy use considering the different task and contextual variables that may affect the differential activation of these strategies.

Finally, from an educational standpoint, evaluating undergraduates’ strategic writing profiles can be important in several ways. Knowing students’ writing strategies will allow teachers to propose writing-to-learn tasks tailored to students’ abilities, which may help reduce the cognitive load of writing and may therefore have a positive impact on students’ domain learning (Torrance and Galbraith, 2006; Kieft et al., 2008). In addition, evaluating undergraduates’ writing strategies will allow identification of poor or poorly defined strategic profiles, which may underlie some students’ writing difficulties (Kieft et al., 2006; Boscolo et al., 2007; Mateos and Solé, 2009; Cumming et al., 2016; Konstantinidou et al., 2023) and complicate how they cope with and succeed in writing tasks. In turn, this will guide the design and implementation of instructional programs that promote students’ strategic writing development (MacArthur et al., 2015; Graham and Harris, 2018; MacArthur and Philippakos, 2022). Thus, the WSQ-SU may be a useful tool that can help give teachers information about their students’ strategies and consequently adapt writing tasks to the students’ writing profiles or help them to adapt writing instruction to their students’ needs. In any case, knowing and considering students’ strategic writing profiles will encourage their academic achievement, as well as their learning and mastery of writing—key elements in academic success, proper socio-personal adjustment, and successful professional careers. In relation to this last point, our study looked at undergraduates studying for degrees in Education, Psychology and Pedagogy. Their professional lives will, in many cases, be aimed at teaching writing. Given those professional profiles, it is even more important and interesting to understand and optimise their writing skills and strategies, since the characteristics or internal variables of future teachers, including their strategic profiles, could mediate their future professional practice when they are teaching novice writers (Graham, 2018; Sánchez-Rivero et al., 2021; Wang and Troia, 2023).

## Data availability statement

The raw data supporting the conclusions of this article will be made available by the authors, without undue reservation.

## Ethics statement

The requirement of ethical approval was waived by Comité de ética University of León for the studies involving humans. The studies were conducted in accordance with the local legislation and institutional requirements. All students agreed to participate voluntarily after verbal informed consent was obtained.

## Author contributions

OA-G: Formal analysis, Methodology, Writing – original draft, Writing – review & editing. PR: Conceptualization, Funding acquisition, Investigation, Writing – original draft, Writing – review & editing.
