# Supplementary material for: An analysis of the psychometric properties of the writing-specific cognitive strategies questionnaire for undergraduate students
Source: Front Psychol. 2023 Nov 16;14:1274478. doi: 10.3389/fpsyg.2023.1274478 (PMC10690414; doi:10.3389/fpsyg.2023.1274478)
Supplement: Supplementary file 1 [file Table_1.DOCX]

Supplementary Material

**The Spanish version of Writing Strategy Questionnaire (Adaptation of Kieft, Rijlaarsdam, & Van den Bergh, 2008)**

A continuación, vas a leer varias afirmaciones sobre diferentes actividades que puedes realizar para escribir un texto. Debes poner tu grado de acuerdo o desacuerdo con cada afirmación según las actividades que tú realizas al escribir. Para ello debes seguir una escala de respuesta que va desde 1: Totalmente en desacuerdo a 5: totalmente de acuerdo. Es muy importante que sepas que las respuestas nunca van a ser incorrectas. Las frases que leerás tan sólo reflejan diferentes formas que cada persona usa para escribir.

|  | |  | Totalmente en desacuerdo | |  | Totalmente de acuerdo |
| --- | --- | --- | --- | --- | --- | --- |
|  | |  | | 1 2 3 4 5 | | |
|  | | Cuando escribo un texto, dedico mucho tiempo a pensar cómo voy a hacerlo. | | 🞏 🞏 🞏 🞏 🞏 | | |
|  | | Siempre realizo un esquema antes de comenzar a escribir. | | 🞏 🞏 🞏 🞏 🞏 | | |
|  | | Antes del texto apunto algunas notas en una hoja en sucio, y luego elaboro mis notas. | | 🞏 🞏 🞏 🞏 🞏 | | |
|  | | Antes de comenzar a escribir un texto, prefiero escribir algunas de mis ideas en una hoja en sucio, para descubrir qué pienso sobre el tema. | | 🞏 🞏 🞏 🞏 🞏 | | |
|  | | Planificar el texto no es útil para mí. | | 🞏 🞏 🞏 🞏 🞏 | | |
|  | | Cuando comienzo a escribir no sé cuál será el contenido de mi texto. | | 🞏 🞏 🞏 🞏 🞏 | | |
|  | | Antes de comenzar a escribir tengo claro qué quiero conseguir en mis lectores. | | 🞏 🞏 🞏 🞏 🞏 | | |
|  | | Mientras estoy escribiendo compruebo cada poco que mi texto no tenga oraciones incorrectas o demasiado largas. | | 🞏 🞏 🞏 🞏 🞏 | | |
|  | | Cuando sé globalmente qué es lo que voy a escribir, escribo mi texto con mucha facilidad. | | 🞏 🞏 🞏 🞏 🞏 | | |
|  | | Cuando escribo un texto, es difícil para mi tener ideas sobre las que escribir. | | 🞏 🞏 🞏 🞏 🞏 | | |
|  | Debo tener mis ideas claras antes de comenzar a escribir. | | | 🞏 🞏 🞏 🞏 🞏 | | |
|  | Cuando escribo un texto, de vez en cuando me pregunto a mí mismo, si mi texto será fácil de comprender para los lectores. | | | 🞏 🞏 🞏 🞏 🞏 | | |
|  | Antes de escribir una oración la tengo clara en mi mente. | | | 🞏 🞏 🞏 🞏 🞏 | | |
|  | Cuando escribo hay veces que escribo párrafos que sé que aún no están correctamente escritos, pero prefiero seguir escribiendo. | | | 🞏 🞏 🞏 🞏 🞏 | | |
|  | Escribir me ayuda a aclarar mis ideas y pensamientos. | | | 🞏 🞏 🞏 🞏 🞏 | | |
|  | Normalmente, los textos que escribo no son muy creativos. | | | 🞏 🞏 🞏 🞏 🞏 | | |
|  | Normalmente entrego mi texto sin comprobar que sus párrafos están bien organizados. | | | 🞏 🞏 🞏 🞏 🞏 | | |
|  | Cuando releo y rescribo mi texto, su estructura cambia mucho. | | | 🞏 🞏 🞏 🞏 🞏 | | |
|  | Antes de entregar mi texto, compruebo si está correctamente estructurado. | | | 🞏 🞏 🞏 🞏 🞏 | | |
|  | Algunas veces, cuando escribo el borrador de mi texto, y una idea no me surge continuo con otra parte de mi texto que me surja, volviendo a la idea anterior más tarde. | | | 🞏 🞏 🞏 🞏 🞏 | | |
|  | Cuando rescribo mis textos, el contenido a menudo cambia mucho. | | | 🞏 🞏 🞏 🞏 🞏 | | |
|  | Cuando releo mis textos, algunas veces son caóticos, liosos. | | | 🞏 🞏 🞏 🞏 🞏 | | |
|  | Tengo que leer los textos que escribo para evitar redundancias, contenidos repetidos. | | | 🞏 🞏 🞏 🞏 🞏 | | |
|  | No presto mucha atención a si estoy satisfecho conmigo mismo por mi texto. | | | 🞏 🞏 🞏 🞏 🞏 | | |
|  | Antes de comenzar a escribir, debo saber cuál será el contenido de mi texto. Por lo tanto, planificar es importante en mi escritura. | | | 🞏 🞏 🞏 🞏 🞏 | | |
|  | Cuando acabo de escribir, releo y mejoro mucho mi texto; puedo cambiar mucho en mi texto. | | | 🞏 🞏 🞏 🞏 🞏 | | |

Muchas gracias por tu colaboración.
